# Supplementary material for: Heparin-based hydrogel scaffolding alters the transcriptomic profile and increases the chemoresistance of MDA-MB-231 triple-negative breast cancer cells
Source: Biomater Sci. 2020 Feb 13;8(10):2786–96. doi: 10.1039/c9bm01481k (PMC7497406; doi:10.1039/c9bm01481k)
Supplement: Supplementary file 2 [file BM-008-C9BM01481K-s002.zip › Supplementary File 4/HP-BhydrogelvControl/Pathways/my_analysis.Gsea.1545201079907/HALLMARK_KRAS_SIGNALING_UP.html]

Details for gene set HALLMARK\_KRAS\_SIGNALING\_UP[GSEA]

|  || Dataset | expr.class.cls#HYD\_versus\_CONTROL.class.cls#HYD\_versus\_CONTROL\_repos |
| Phenotype | class.cls#HYD\_versus\_CONTROL\_repos |
| Upregulated in class | CONTROL |
| GeneSet | HALLMARK\_KRAS\_SIGNALING\_UP |
| Enrichment Score (ES) | -0.29058787 |
| Normalized Enrichment Score (NES) | -1.3386642 |
| Nominal p-value | 0.01904762 |
| FDR q-value | 0.06383315 |
| FWER p-Value | 0.395 |
Table: GSEA Results Summary

  

Fig 1: Enrichment plot: HALLMARK\_KRAS\_SIGNALING\_UP      
 Profile of the Running ES Score & Positions of GeneSet Members on the Rank Ordered List

  

| PROBE | DESCRIPTION (from dataset) | GENE SYMBOL | GENE\_TITLE | RANK IN GENE LIST | RANK METRIC SCORE | RUNNING ES | CORE ENRICHMENT || 1 | TPH1 | na |  |  | 907 | 1.942 | -0.0260 | No |
| 2 | ID2 | na |  |  | 1383 | 1.637 | -0.0327 | No |
| 3 | EPHB2 | na |  |  | 1523 | 1.566 | -0.0226 | No |
| 4 | PPP1R15A | na |  |  | 1561 | 1.546 | -0.0073 | No |
| 5 | ATG10 | na |  |  | 1589 | 1.537 | 0.0083 | No |
| 6 | ANGPTL4 | na |  |  | 1665 | 1.505 | 0.0211 | No |
| 7 | AKT2 | na |  |  | 1769 | 1.459 | 0.0320 | No |
| 8 | CSF2RA | na |  |  | 1803 | 1.441 | 0.0463 | No |
| 9 | ENG | na |  |  | 2136 | 1.298 | 0.0433 | No |
| 10 | CBX8 | na |  |  | 2200 | 1.276 | 0.0542 | No |
| 11 | GADD45G | na |  |  | 2432 | 1.199 | 0.0554 | No |
| 12 | ITGB2 | na |  |  | 2483 | 1.179 | 0.0659 | No |
| 13 | WDR33 | na |  |  | 2704 | 1.113 | 0.0667 | No |
| 14 | GALNT3 | na |  |  | 3491 | 0.920 | 0.0357 | No |
| 15 | IGF2 | na |  |  | 3750 | 0.858 | 0.0317 | No |
| 16 | SEMA3B | na |  |  | 3816 | 0.847 | 0.0377 | No |
| 17 | CD37 | na |  |  | 4878 | 0.645 | -0.0108 | No |
| 18 | BMP2 | na |  |  | 4891 | 0.643 | -0.0043 | No |
| 19 | ITGBL1 | na |  |  | 4939 | 0.636 | 0.0003 | No |
| 20 | DOCK2 | na |  |  | 4960 | 0.634 | 0.0063 | No |
| 21 | LIF | na |  |  | 5094 | 0.614 | 0.0062 | No |
| 22 | RGS16 | na |  |  | 5502 | 0.552 | -0.0090 | No |
| 23 | SCG5 | na |  |  | 5507 | 0.551 | -0.0031 | No |
| 24 | HBEGF | na |  |  | 5567 | 0.542 | -0.0002 | No |
| 25 | LAT2 | na |  |  | 5610 | 0.537 | 0.0036 | No |
| 26 | TNFRSF1B | na |  |  | 5672 | 0.529 | 0.0063 | No |
| 27 | MAFB | na |  |  | 5745 | 0.520 | 0.0083 | No |
| 28 | PTPRR | na |  |  | 6285 | 0.446 | -0.0151 | No |
| 29 | ANO1 | na |  |  | 6311 | 0.443 | -0.0114 | No |
| 30 | PTGS2 | na |  |  | 6325 | 0.440 | -0.0072 | No |
| 31 | SNAP25 | na |  |  | 6453 | 0.422 | -0.0092 | No |
| 32 | TLR8 | na |  |  | 6558 | 0.409 | -0.0101 | No |
| 33 | CXCL10 | na |  |  | 6890 | 0.372 | -0.0233 | No |
| 34 | TNFAIP3 | na |  |  | 6980 | 0.364 | -0.0240 | No |
| 35 | KLF4 | na |  |  | 7185 | 0.341 | -0.0309 | No |
| 36 | IL1RL2 | na |  |  | 7318 | 0.323 | -0.0342 | No |
| 37 | RBM4 | na |  |  | 7336 | 0.321 | -0.0315 | No |
| 38 | ALDH1A2 | na |  |  | 8078 | 0.248 | -0.0677 | No |
| 39 | TRAF1 | na |  |  | 8359 | 0.212 | -0.0800 | No |
| 40 | CROT | na |  |  | 8403 | 0.207 | -0.0799 | No |
| 41 | DNMBP | na |  |  | 8630 | 0.188 | -0.0897 | No |
| 42 | MAP7 | na |  |  | 8695 | 0.182 | -0.0911 | No |
| 43 | GPNMB | na |  |  | 8740 | 0.175 | -0.0914 | No |
| 44 | PCSK1N | na |  |  | 8793 | 0.167 | -0.0923 | No |
| 45 | HKDC1 | na |  |  | 8863 | 0.157 | -0.0941 | No |
| 46 | SCN1B | na |  |  | 9124 | 0.137 | -0.1063 | No |
| 47 | IL1B | na |  |  | 9136 | 0.136 | -0.1053 | No |
| 48 | BTBD3 | na |  |  | 9187 | 0.128 | -0.1065 | No |
| 49 | PRDM1 | na |  |  | 9378 | 0.106 | -0.1153 | No |
| 50 | CMKLR1 | na |  |  | 9419 | 0.102 | -0.1163 | No |
| 51 | CDADC1 | na |  |  | 9426 | 0.100 | -0.1155 | No |
| 52 | ANKH | na |  |  | 9443 | 0.098 | -0.1152 | No |
| 53 | CCND2 | na |  |  | 9609 | 0.088 | -0.1229 | No |
| 54 | ALDH1A3 | na |  |  | 9613 | 0.087 | -0.1221 | No |
| 55 | PRKG2 | na |  |  | 9669 | 0.078 | -0.1241 | No |
| 56 | FUCA1 | na |  |  | 9835 | 0.060 | -0.1321 | No |
| 57 | ADAM8 | na |  |  | 9924 | 0.050 | -0.1362 | No |
| 58 | TSPAN1 | na |  |  | 10112 | 0.027 | -0.1457 | No |
| 59 | ETV4 | na |  |  | 10161 | 0.020 | -0.1480 | No |
| 60 | CFB | na |  |  | 10199 | 0.015 | -0.1498 | No |
| 61 | TRIB2 | na |  |  | 10397 | 0.007 | -0.1600 | No |
| 62 | MAP3K1 | na |  |  | 10425 | 0.004 | -0.1614 | No |
| 63 | GPRC5B | na |  |  | 10648 | -0.023 | -0.1728 | No |
| 64 | RABGAP1L | na |  |  | 10753 | -0.033 | -0.1779 | No |
| 65 | AVL9 | na |  |  | 11060 | -0.073 | -0.1931 | No |
| 66 | JUP | na |  |  | 11065 | -0.073 | -0.1925 | No |
| 67 | ADAM17 | na |  |  | 11082 | -0.075 | -0.1925 | No |
| 68 | KCNN4 | na |  |  | 11086 | -0.076 | -0.1918 | No |
| 69 | PTBP2 | na |  |  | 11362 | -0.098 | -0.2052 | No |
| 70 | NIN | na |  |  | 11437 | -0.106 | -0.2079 | No |
| 71 | PLAUR | na |  |  | 11509 | -0.112 | -0.2104 | No |
| 72 | PLAU | na |  |  | 11513 | -0.113 | -0.2093 | No |
| 73 | YRDC | na |  |  | 11535 | -0.117 | -0.2091 | No |
| 74 | RELN | na |  |  | 11544 | -0.118 | -0.2082 | No |
| 75 | SDCCAG8 | na |  |  | 11692 | -0.137 | -0.2144 | No |
| 76 | G0S2 | na |  |  | 11856 | -0.159 | -0.2212 | No |
| 77 | FBXO4 | na |  |  | 11927 | -0.166 | -0.2230 | No |
| 78 | CTSS | na |  |  | 11932 | -0.167 | -0.2213 | No |
| 79 | TMEM158 | na |  |  | 12241 | -0.205 | -0.2352 | No |
| 80 | MTMR10 | na |  |  | 12287 | -0.211 | -0.2352 | No |
| 81 | ETS1 | na |  |  | 12310 | -0.215 | -0.2340 | No |
| 82 | SPARCL1 | na |  |  | 12400 | -0.222 | -0.2362 | No |
| 83 | F13A1 | na |  |  | 12652 | -0.241 | -0.2467 | No |
| 84 | CCL20 | na |  |  | 12777 | -0.255 | -0.2504 | No |
| 85 | VWA5A | na |  |  | 12806 | -0.258 | -0.2490 | No |
| 86 | HDAC9 | na |  |  | 12887 | -0.270 | -0.2501 | No |
| 87 | CFH | na |  |  | 12948 | -0.278 | -0.2502 | No |
| 88 | CSF2 | na |  |  | 13029 | -0.288 | -0.2512 | No |
| 89 | AKAP12 | na |  |  | 13048 | -0.290 | -0.2489 | No |
| 90 | PECAM1 | na |  |  | 13230 | -0.314 | -0.2549 | No |
| 91 | GFPT2 | na |  |  | 13386 | -0.335 | -0.2593 | No |
| 92 | CAB39L | na |  |  | 13466 | -0.348 | -0.2596 | No |
| 93 | INHBA | na |  |  | 13523 | -0.357 | -0.2586 | No |
| 94 | NRP1 | na |  |  | 13560 | -0.362 | -0.2564 | No |
| 95 | GNG11 | na |  |  | 13602 | -0.369 | -0.2545 | No |
| 96 | PLAT | na |  |  | 13867 | -0.414 | -0.2637 | No |
| 97 | PTCD2 | na |  |  | 14049 | -0.424 | -0.2685 | No |
| 98 | TRIB1 | na |  |  | 14135 | -0.432 | -0.2682 | No |
| 99 | MMP11 | na |  |  | 14247 | -0.452 | -0.2690 | No |
| 100 | LAPTM5 | na |  |  | 14652 | -0.515 | -0.2844 | No |
| 101 | SATB1 | na |  |  | 14770 | -0.532 | -0.2847 | Yes |
| 102 | EVI5 | na |  |  | 14792 | -0.536 | -0.2798 | Yes |
| 103 | CPE | na |  |  | 14839 | -0.543 | -0.2762 | Yes |
| 104 | USP12 | na |  |  | 15065 | -0.573 | -0.2816 | Yes |
| 105 | SPRY2 | na |  |  | 15106 | -0.581 | -0.2773 | Yes |
| 106 | SNAP91 | na |  |  | 15228 | -0.601 | -0.2769 | Yes |
| 107 | AMMECR1 | na |  |  | 15247 | -0.606 | -0.2711 | Yes |
| 108 | PSMB8 | na |  |  | 15412 | -0.636 | -0.2727 | Yes |
| 109 | CBR4 | na |  |  | 15419 | -0.636 | -0.2659 | Yes |
| 110 | IL7R | na |  |  | 15528 | -0.657 | -0.2643 | Yes |
| 111 | PDCD1LG2 | na |  |  | 15650 | -0.676 | -0.2631 | Yes |
| 112 | TSPAN7 | na |  |  | 15654 | -0.677 | -0.2557 | Yes |
| 113 | ZNF639 | na |  |  | 15662 | -0.679 | -0.2485 | Yes |
| 114 | STRN | na |  |  | 15691 | -0.680 | -0.2425 | Yes |
| 115 | MPZL2 | na |  |  | 15948 | -0.732 | -0.2477 | Yes |
| 116 | CBL | na |  |  | 16094 | -0.768 | -0.2468 | Yes |
| 117 | IGFBP3 | na |  |  | 16184 | -0.784 | -0.2428 | Yes |
| 118 | TSPAN13 | na |  |  | 16213 | -0.790 | -0.2354 | Yes |
| 119 | HIST1H2BB | na |  |  | 16471 | -0.847 | -0.2395 | Yes |
| 120 | ST6GAL1 | na |  |  | 16507 | -0.856 | -0.2318 | Yes |
| 121 | TFPI | na |  |  | 16601 | -0.872 | -0.2270 | Yes |
| 122 | SOX9 | na |  |  | 16805 | -0.929 | -0.2273 | Yes |
| 123 | PRRX1 | na |  |  | 16827 | -0.933 | -0.2180 | Yes |
| 124 | F2RL1 | na |  |  | 16911 | -0.960 | -0.2117 | Yes |
| 125 | EREG | na |  |  | 17124 | -1.024 | -0.2115 | Yes |
| 126 | TOR1AIP2 | na |  |  | 17151 | -1.031 | -0.2014 | Yes |
| 127 | ITGA2 | na |  |  | 17270 | -1.074 | -0.1956 | Yes |
| 128 | BTC | na |  |  | 17306 | -1.086 | -0.1854 | Yes |
| 129 | BIRC3 | na |  |  | 17468 | -1.140 | -0.1811 | Yes |
| 130 | DCBLD2 | na |  |  | 17598 | -1.196 | -0.1746 | Yes |
| 131 | ETV5 | na |  |  | 17798 | -1.286 | -0.1707 | Yes |
| 132 | ETV1 | na |  |  | 17920 | -1.340 | -0.1622 | Yes |
| 133 | MALL | na |  |  | 17971 | -1.363 | -0.1496 | Yes |
| 134 | PLEK2 | na |  |  | 18051 | -1.414 | -0.1381 | Yes |
| 135 | NGF | na |  |  | 18094 | -1.437 | -0.1243 | Yes |
| 136 | DUSP6 | na |  |  | 18261 | -1.536 | -0.1159 | Yes |
| 137 | MMD | na |  |  | 18291 | -1.558 | -0.1001 | Yes |
| 138 | GLRX | na |  |  | 18498 | -1.703 | -0.0920 | Yes |
| 139 | BPGM | na |  |  | 18542 | -1.737 | -0.0749 | Yes |
| 140 | ZNF277 | na |  |  | 18573 | -1.760 | -0.0569 | Yes |
| 141 | EMP1 | na |  |  | 18689 | -1.884 | -0.0420 | Yes |
| 142 | HSD11B1 | na |  |  | 18701 | -1.893 | -0.0215 | Yes |
| 143 | CXCR4 | na |  |  | 18827 | -2.050 | -0.0053 | Yes |
| 144 | ANXA10 | na |  |  | 18925 | -2.219 | 0.0143 | Yes |
Table: GSEA details [plain text format]

  

Fig 2: HALLMARK\_KRAS\_SIGNALING\_UP      
 Blue-Pink O' Gram in the Space of the Analyzed GeneSet

  

Fig 3: HALLMARK\_KRAS\_SIGNALING\_UP: Random ES distribution      
 Gene set null distribution of ES for **HALLMARK\_KRAS\_SIGNALING\_UP**
